# Detecting and Visualizing Stops in Dance Training by Neural Network Based on Velocity and Acceleration

**DOI:** 10.3390/s22145402

**Published:** 2022-07-20

**Authors:** Yuuki Jin, Genki Suzuki, Hiroyuki Shioya

**Affiliations:** Division of Information and Electronic Engineering, Muroran Institute of Technology, Muroran 050-8585, Japan; kokoronomotono@gmail.com (Y.J.); shioya@mmm.muroran-it.ac.jp (H.S.)

**Keywords:** neural network, motion sensor, dance analysis, data visualization

## Abstract

Various genres of dance, such as Yosakoi Soran, have contributed to the health of many people and contributed to their sense of belonging to a community. However, due to the effects of COVID-19, various face-to-face activities have been restricted and group dance practice has become difficult. Hence, there is a need to facilitate remote dance practice. In this paper, we propose a system for detecting and visualizing the very important dance motions known as stops. We measure dance movements by motion capture and calculate the features of each movement based on velocity and acceleration. Using a neural network to learn motion features, the system detects stops and visualizes them using a human-like 3D model. In an experiment using dance data, the proposed method obtained highly accurate stop detection results and demonstrated its effectiveness as an information and communication technology support for remote group dance practice.

## 1. Introduction

There are various genres of dance around the world, such as ballet, hip hop, and Japanese dance. Like sports, dance is popular for its strong exercise benefit [1,2,3,4,5,6,7]. Dance also has artistic aspects linked to the culture of a region and country, and it is effective for revitalizing the community of a local region [8,9]. To perfect dance techniques, it is essential for a performer to repeatedly practice choreography composed specifically for a song. In general, the performer practices by getting advice on choreography from an instructor and watching sample videos of the choreography [10,11]. However, it is challenging to practice face-to-face because of the spread of COVID-19 infection in recent years. In addition, both the number of instructors and the time available for practice are limited. However, information and communication technology (ICT) and artificial intelligence (AI) technologies can now be used to develop techniques to improve the efficiency of dance practice.

**Figure 1:**
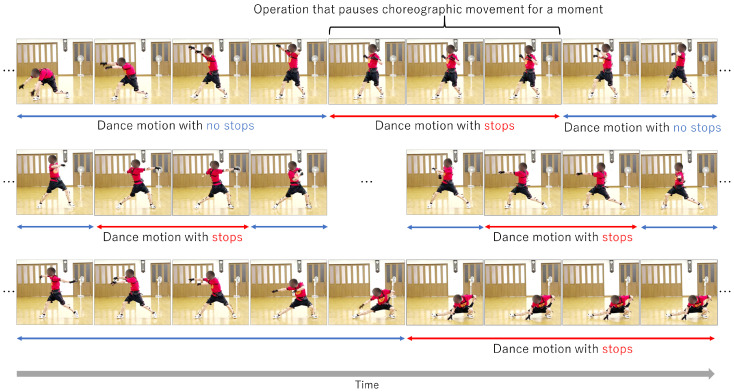
Demonstration of flow of motion in Yosakoi Soran dance. From left to right in the first line: no stop, stops, no stop. The stops factor is incorporated into the continuous dance process as a mid-period.

The Yosakoi Soran Festival is a typical Japanese dance event held in Hokkaido. This annual event has an economic effect of more than 20 billion yen (around $173 million USD) because many tourists visit it (https://app.yosakoi-soran.jp/news/view/324, accessed on 16 March 2022). Since Yosakoi Soran [12] is a group dance in which ordinary citizens participate, it is important not only to choreograph individual dances but also to synchronize the troupe members’ movements. In particular, abrupt stops during a dance are important in choreography and are especially important for members of a troupe to synchronize with each other. Abrupt stops are an important component not only of Yosakoi Soran but also in traditional Asian dances in China, India, and elsewhere. Figure 1 shows how a stop is performed. Although synchronization of stops among troupe members is key to the successful performance of Yosakoi Soran, it is difficult for performers to notice slight differences in timing between themselves and the other performers. Therefore, it is necessary to construct a dance practice system that detects the timing of stops in a remote environment or in an on-demand format. Our proposal supports individual stopping practice and thus can contribute to improving dance skills.

Various researchers have recently proposed analyses of dance movements using motion capture (MoCap) for teaching and training [13,14,15,16,17,18,19,20]. Those analyses have focused on the performer’s pose during a dance. In [13,14], a dance practice support system using MoCap was developed. MoCap-based systems obtain the dance movements of experts and nonexpert performers, calculate the differences between the two, and feed those differences back to the latter. These studies show the effectiveness of using MoCap to support dance practice. In addition, it has been reported that performers prefer dance practices based on an interactive system rather than conventional practice methods such as watching choreography videos and direct instruction by instructors [21]. For Yosakoi Soran in particular, an interactive practice system must help performers synchronize their starts and stops with those of the other troupe members. The importance of synchronized stops during a group dance is shown in [22]. Therefore, it is necessary to analyze the velocities of the joints of a performer during a dance. Moreover, since the choreography of a stop involves the sharpness of movement, analysis focusing on acceleration is also important.

**Figure 2:**
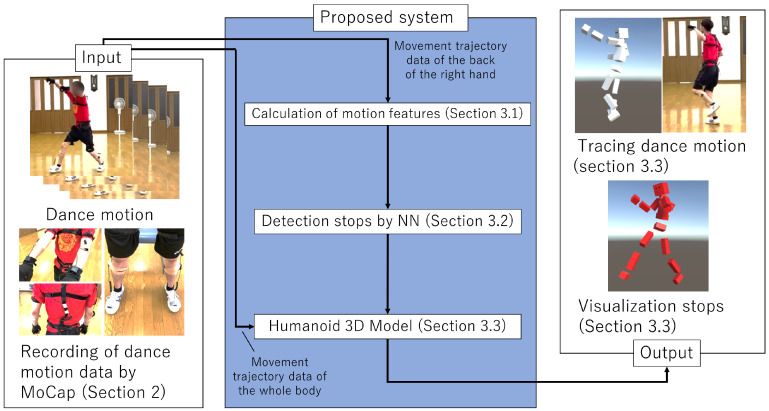
Overview of the proposed system. The dance motion data are recorded by MoCap (Section 2). The proposed system consists of three phases (Section 3). First, the motion features based on velocity and acceleration are calculated (Section 3.1). Stops are detected by a neural network model (Section 3.2). Stops are visualized using a humanoid 3D model via virtual reality spaces (Section 3.3).

This paper proposes a new stop detection method based on a neural network using a motion sensor’s velocity and the acceleration of joint position. An overview of the proposed method is shown in Figure 2. First, the motion data of a Yosakoi Soran performer is obtained by using MoCap. Next, motion features based on velocity and acceleration are calculated from the time-series data of the 3D coordinates of the right fist. Then, a stop is detected based on a feedforward neural network (NN) [23] using those features. Finally, the detected stops are visualized with a 3D humanoid model. The proposed method contributes to the automatic evaluation of Yosakoi Soran movements because it detects the choreographed timing of stops. In addition to streamlining individual repetition practice for Yosakoi Soran, this method is expected to be adapted for various other dance forms that use stops.

This paper is organized as follows. In Section 2, the measurement method of the dance movement by MoCap is explained. In Section 3, the proposed method is explained. Specifically, the method for calculating features based on velocity and acceleration from dance motions and the method for visualizing detected stops are described. In Section 4, the experiments are described. Specifically, the effectiveness of the proposed method is shown by comparing the accuracy of the detection and the visualization of stops between the proposed method and methods for comparison. In Section 5, the conclusions of this paper and future work are described.

## 2. Recording of Dance Motion Data by MoCap

**Figure 3:**
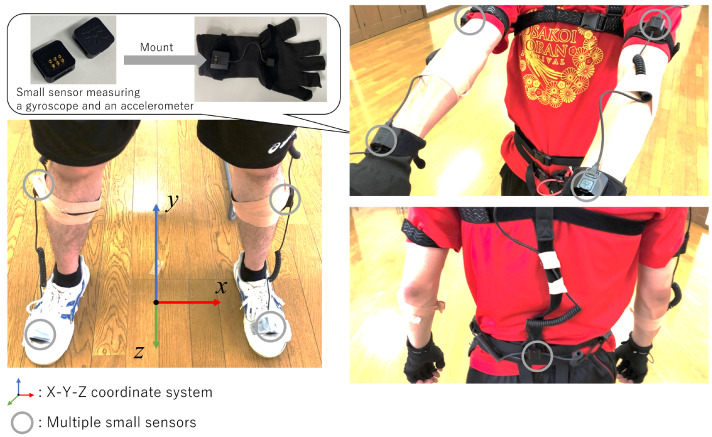
Attachment of PN to the performer is presented with 18 small sensors at hand, arm, shoulder, leg, head, and waist that measure inertia, such as a gyroscope and an accelerometer. The relative positions among the sensors are measured, and the 3D positions of the sensors are obtained.

In this section, we explain how to record a performer’s motion data by MoCap to detect stops in Yosakoi Soran. Using Perception Neuron 2.0 (https://www.noitom.com/, accessed on 16 March 2022) (PN), which is NOITOM’s motion capture shown in Figure 3, the performer’s dance motion is recorded while the performer wears the MoCap. The MoCap consists of multiple small sensors that measure inertia, such as a gyroscope and an accelerometer. Based on the data acquired from these sensors, the posture and position of the performer are estimated.

There are two main advantages of PN. While PN is less accurate than optical MoCaps in estimating posture and position, PN costs only about 1/100 to 1/1000 the cost of an optical MoCap. From this point of view, the practical application of the proposed system becomes realistic. The other advantage of the proposed system is that it has fewer restrictions on its use. For example, an optical MoCap is highly accurate but requires a dedicated studio with multiple cameras and can measure motion only within a limited area. In contrast, PN can be used anywhere if nearby metal or magnetic objects are kept away. On the other hand, PN has some disadvantages: over a long period, errors in a PN gradually increase due to problems in posture- and position-estimation methods. Moreover, the accuracy of position estimation is low. However, if the PN is calibrated periodically and used in a large space where the influence of electromagnetic waves is minimized, the error can be minimized to the extent possible. Actually, Yosakoi musical pieces are short (about 3–10 min each), so the effect of errors caused by continuous use is considered small. Instead, PN is used to analyze various movements such as surgical simulation [24] and the analysis of sports movements [25]. Within this background, we adapt PN for our analysis of dance movement.

In dance, the part of the body that has the widest range of movement is the hand. Therefore, in this study, as shown in Figure 3, the MoCap is attached to the performer’s hand, and the dance movement, including stops, is recorded. This study describes a method of detecting stops using the movement trajectory data on 3D coordinates of the back of the right hand among the recorded data.

## 3. Detection and Visualization of Stops by NN Based on Velocity and Acceleration

This section describes the proposed method of detecting stops based on NN and the visualization of detected stops. First, the motion features consist of velocities, and acceleration is calculated using the movement trajectory data of the back of the right hand as recorded by the MoCap. Section 3.1 describes the method of calculating the motion features of a stop. Then, the detection model is constructed based on NN using those features in Section 3.2, and Section 3.3 describes the method of visualizing the detected stops. The details are shown below.

### 3.1. Calculation of Motion Features

Since stopping is an operation that pauses choreographic movement for a moment, the characteristics of the velocity and acceleration of the hand are calculated. First, we define the movement trajectory data $f_{i,j}\left(i=1,2,\ldots ,N;j\in \left\{x,y,z\right\}\right|N$ is the number of series of movement loci) of the back of the right hand from MoCap.

#### 3.1.1. Calculation of Velocity

Since the sensor mounted on the MoCap is extremely sensitive, the obtained movement trajectory data contain high-frequency components such as noise. It is desirable to apply smoothing to the movement trajectory data to remove noise in advance because the calculation of velocity and acceleration requires differentiation of the movement trajectory data. Therefore, the moving average processing [26] is applied to $f_{i,j}$, as shown in Equation (1).

(1) $$f_{i,j}^{\mathrm{ma}}=\frac{1}{2n+1}\sum_{k=i-n}^{i+n}f_{k,j},$$

where, *n* is an arbitrary natural number. Note that *n* and the sampling rate of $f_{k,j}$ were empirically set to 5 (i.e., window size being 12) and 60 Hz, respectively, based on prior equipment preparation for data acquisition. Then, the velocity $f_{i,j}^{\prime }$ is calculated by the 5-point approximation formula of the derivative in Equation (2).

(2) $$f_{i,j}^{\prime }=\frac{f_{i-2h,j}^{\mathrm{ma}}-8f_{i-h,j}^{\mathrm{ma}}+8f_{i+h,j}^{\mathrm{ma}}-f_{i+2h,j}^{\mathrm{ma}}}{12h},$$

where, although *h* is a minute width of differentiation, h=1 is set to obtain the displacement for each frame in this paper. If a high-frequency component is also generated in the calculated velocity $f_{i,j}^{\prime }$, the velocity $f_{i,j}^{\prime }$ is applied to the subsequent calculation of the acceleration. Therefore, it is necessary to smooth the transition of $f_{i,j}^{\prime }$. However, the velocity $f_{i,j}^{\prime }$ may change drastically, and if moving average processing is applied, the size characteristics may be impaired. Therefore, the following root mean square $f_{i,j}^{\mathrm{rms}}$ is calculated from the velocity $f_{i,j}^{\prime }$ in Equation (3).

(3) $$f_{i,j}^{\mathrm{rms}}=\sqrt{\frac{1}{2n+1}\sum_{k=i-n}^{i+n}{\left(f_{k,j}^{\prime }\right)}^{2}}.$$

Then, the one-dimensional velocity $f_{i}^{\mathrm{abs}}$ is calculated from the three-dimensional velocity as shown in Equation (4).

(4) $$f_{i}^{\mathrm{abs}}=\sqrt{{\left(f_{i,x}^{\mathrm{rms}}\right)}^{2}+{\left(f_{i,y}^{\mathrm{rms}}\right)}^{2}+{\left(f_{i,z}^{\mathrm{rms}}\right)}^{2}}.$$

**Figure 4:**
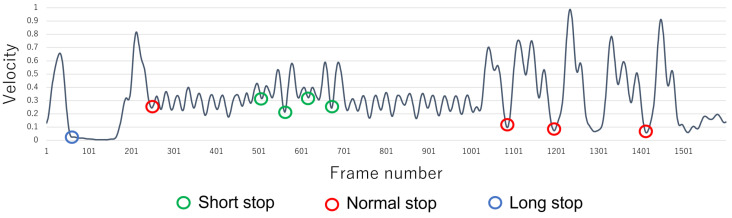
Example of velocity transition.

Finally, the change in velocity $f_{i}^{\mathrm{abs}}$ is smoothed by reapplying Equation (2) to the result obtained in Equation (4). Note that the velocity $v_{i}^{\mathrm{norm}}$ is calculated by normalization in the range of 0 to 1. Moreover, the minimum and maximum values used in normalization are determined independently for each set of motion data acquired from each subject. An example of the calculated velocity $v_{i}^{\mathrm{norm}}$ is shown in Figure 4. The green, red, and blue circles indicate frames that have been visually confirmed to be stopped. It can be confirmed that some of the frames whose velocities are minimal or approach 0 are stops (frames of stop possibility, i.e., of the possibility of stopped motion). The green, red, and blue circles are defined as short, normal, and long stops, respectively. Moreover, among the stop possibility frames, a frame that shows no stop is defined as no stop. Table 1 shows the characteristics of each stop. However, since the characteristics of short, normal, and long stops differ, they are labeled separately to improve the NN’s accuracy of stop detection below.

#### 3.1.2. Calculation of Acceleration

To detect stopping, we calculate acceleration, which is strongly related to the sharpness of movements. Specifically, using the calculated velocity $v_{i}^{\mathrm{norm}}$, the first derivative $a_{i}$ of the velocity $v_{i}^{\mathrm{norm}}$ is calculated by the 5-point approximation formula, as shown in Equation (5).

(5) $$a_{i}=\frac{v_{i-2h}^{\mathrm{norm}}-8v_{i-h}^{\mathrm{norm}}+8v_{i+h}^{\mathrm{norm}}-v_{i+2h}^{\mathrm{norm}}}{12h},$$

where, to obtain the displacement for each frame, h=1 is set in this paper. Then, $a_{i}^{\mathrm{norm}}$ is calculated by normalizing the result obtained by Equation (5) in the range of −1 to 1, and the minimum and maximum values are determined the same way as the calculation of $v_{i}^{\mathrm{norm}}$. At this time, the frame at the moment when $a_{i}^{\mathrm{norm}}$ becomes a value from negative to zero or more is the frame when velocity $v_{i}^{\mathrm{norm}}$ becomes the minimum. However, the frame at the moment when the velocity $v_{i}^{\mathrm{norm}}$ approaches zero is slightly delayed with this method. Therefore, the acceleration $a_{i}^{\mathrm{fil}}$ is calculated from $a_{i}^{\mathrm{norm}}$ as shown in Equation (6).

(6) $$a_{i}^{\mathrm{fil}}=\left\{\begin{array}{cc} 0 & \mathrm{if}\;-0.05\leq a_{i}^{\mathrm{norm}}\leq 0.05,\\ a_{i}^{\mathrm{norm}} & \mathrm{else}. \end{array}\right.$$

**Figure 5:**
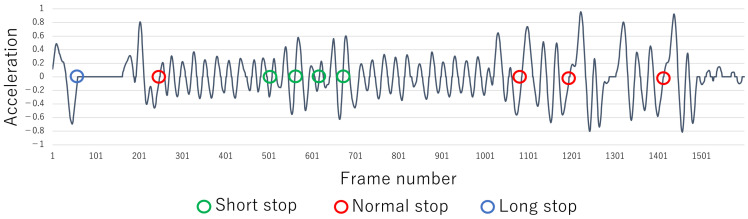
Example of acceleration transition.

Note that the threshold (=0.05) is set to suppress minute discrepancies, determined empirically from test data from multiple experiments. By calculating $a_{i}^{\mathrm{fil}}$, it is possible to find all stop possibility frames. An example of the acceleration $a_{i}^{\mathrm{fil}}$ is shown in Figure 5. The green, red, and blue circles are frames whose accelerations change from negative to 0 and which correspond to each stop or stop possibility frame in Figure 4.

#### 3.1.3. Construction of Training Data Set for Stop Detection

The NN’s training data are constructed by using velocity and acceleration. The frame number of each stop possibility frame in Figure 4 and Figure 5 is called *t*. In addition, the operation’s stop time tended to be about 25 to 50 frames. Therefore, the characteristics related to before and after the stop possibility frame *t* are calculated from Equations (7)–(10) by using $v_{i}^{\mathrm{norm}}$ and $a_{i}^{\mathrm{fil}}$. As a result, the NN’s training data for four elements $r_{t}=[V_{\mathrm{back}}\left(t\right),V_{\mathrm{forward}}\left(t\right),$$A_{\mathrm{back}}\left(t\right),A_{\mathrm{forward}}{\left(t\right)]}^{T}$ are obtained. These are approximations of the graph area of Figure 4 and Figure 5 in the range of 25 frames before and after the *t* frame.

(7) $$\begin{array}{ccc} V_{\mathrm{back}}\left(t\right) & = & \frac{1}{2}\sum_{k=t-25}^{t-1}\left(v_{k}^{\mathrm{norm}}+v_{k+1}^{\mathrm{norm}}\right), \end{array}$$

(8) $$\begin{array}{ccc} V_{\mathrm{forward}}\left(t\right) & = & \frac{1}{2}\sum_{k=t}^{t+24}\left(v_{k}^{\mathrm{norm}}+v_{k+1}^{\mathrm{norm}}\right), \end{array}$$

(9) $$\begin{array}{ccc} A_{\mathrm{back}}\left(t\right) & = & \frac{1}{2}\sum_{k=t-25}^{t}\left(a_{k}^{\mathrm{fil}}+a_{k+1}^{\mathrm{fil}}\right), \end{array}$$

(10) $$\begin{array}{ccc} A_{\mathrm{forward}}\left(t\right) & = & \frac{1}{2}\sum_{k=t}^{t+25}\left(a_{k}^{\mathrm{fil}}+a_{k+1}^{\mathrm{fil}}\right). \end{array}$$

### 3.2. Construction of a Stop Detection Model of the NN

This section describes how to detect a stop by forwarding the propagation type of the NN using the motion features calculated in the previous section. The NN used in the proposed method consists of an input layer, a hidden N-layer, and an output layer. The motion features of each frame calculated in the previous section are used as a vector with $r_{t}$ as input data. In the output, the values c=1,⋯,C of the class indicating the detection result are associated. Let the number of nodes in the input, hidden, and output layers be M,L, and *C*, respectively. The bias is set by $x_{0}=1$. Let the vector of motion features $r_{t}$ correspond to $\left(x_{1},\cdots ,x_{M}\right)$. The following propagation equation is obtained at the node of the *l* in the middle layer, as shown in Equation (11).

(11) $$h_{l}\left(r_{t}\right)=h\left(\sum_{m=0}^{M}w_{l,m}^{1}x_{m}\right),$$

where, $w_{m,l}^{1}$ is the weight between the input layer and the hidden layer. Note that the rectified linear unit (RELU) function [27] is used as the activation function h(·). The linear sum $y_{c}\left(r_{t}\right)$ of the output of the hidden element, including the bias and the weight $w_{l,c}^{2}$ between the hidden and output layers, is obtained as shown in Equation (12). $y_{c}\left(r_{t}\right)$ is the *c*-th output value in the output layer.

(12) $$y_{c}\left(r_{t}\right)=\sum_{l=0}^{L}w_{c,l}^{2}h_{l}\left(r_{t}\right).$$

Moreover, the following $s_{c}$ can be obtained as the probability value indicating the class by conversion with Equation (13) (softmax function [28]) using all the outputs in the output layer.

(13) $$s_{c}=\frac{\exp\{y_{c}\left(r_{t}\right)\}}{\sum_{c^{\prime }=1}^{C}\exp\left\{y_{c^{\prime }}\left(r_{t}\right)\right\}}.$$

In the NN’s training, the weights and biases for each layer that minimize the cross-entropy error [29] are determined by using the training data set created in the previous section. Finally, the short, normal, long, and no stops are classified by inputting the same test data as the training data format to the trained NN.

### 3.3. Visualization of a Stop by Human-like 3D Model in Virtual Reality

This section describes the visualization of a stop by the proposed system. The system described below was developed using Unity (https://unity.com, accessed on 16 March 2022), a virtual reality (VR) development environment. Unity is used to visualize motion analysis research [20,30] and is also useful for actual application development. So, we used the development engine of this virtual environment. For effective practice, it is important for the system to visualize the differences in stop timing between the expert and the performer. Our proposed system can easily confirm the detection results, side by side, of stops made by the expert and by the performer. In the following sections, we describe the functions of the visualization system in detail.

**Figure 6:**
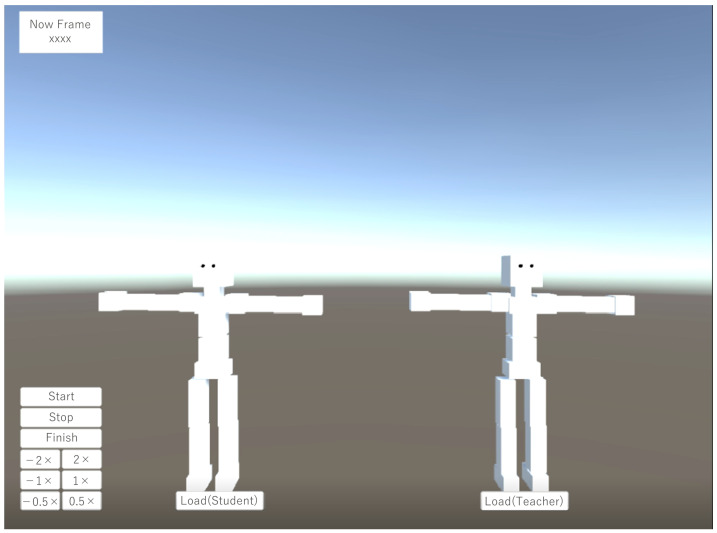
3D model for stop visualization system.

**Figure 7:**
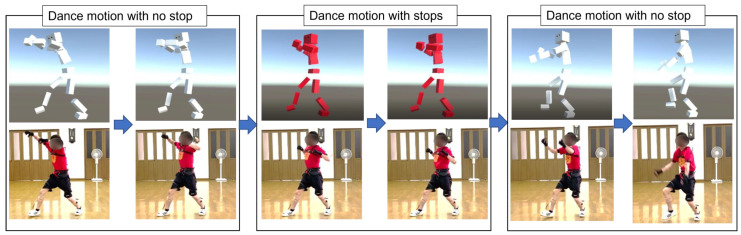
Motion data playback.

First, using the stop detection model constructed in the previous section, we obtain the stop detection results from the motion data for testing. Next, we prepare the two human 3D models shown in Figure 6 in the VR space and adapt the dance motion. By clicking the Load button below the 3D model, the recorded dance motion is applied to each 3D model. By adopting the dance motion to the human 3D model, the model moves in the same way as the dance motion recorded by motion capture and the color of the model changes to red only when a stop is performed during the dance movement, as shown in Figure 7. The details of the various user interfaces (UI) of the visualization system are shown in Table 2. Specifically, the UI allows the user to fast-forward and rewind the motion data at 0.5×, 1×, and 2× speeds. The upper-right corner of the screen displays the current frame number, giving the performer an idea of the timing of the choreography he or she wants to check. One of them is the dance movement of the performer, and the other is the dance movement of the expert. This allows the performer to visually learn the difference in posture and timing of the stops of the performer and the expert.

## 4. Experimental Section

This section verifies the effectiveness of stop detection by the proposed method. It also verifies the visualization accuracy of detected stops.

### 4.1. Verification of Stop Detection Accuracy

In this section, the accuracy of the proposed method in detecting stops is verified by using comparative methods.

#### 4.1.1. Verification of the Effectiveness of the Proposed Method

In this experiment, the stop detection accuracy is verified. First, the data used in this experiment are described. To construct a high-quality dataset of Yosakoi Soran motion in the first 90 s of the song "Yochore", a total of five dance movements performed by three experienced dancers were recorded and used in the experiment. The details of each dance movement and about the three expert dancers (Subject A–Subject C) are shown in Table 3. In the experiment, we confirmed that stable measurement was achieved with data acquired multiple times by calibrating the PN in a space free of electromagnetic radiation. The choreography of “Yochore” used in the experiment is available on YouTube (https://www.youtube.com/watch?v=FRMOpCPw2xA&t=0s, accessed on 16 March 2022). The number of expert performers was set to an appropriate value based on the [13,14,15,17,20,31], which performed the motion analysis. The choreography of the Yosakoi dance was composed for ordinary people to earn and reproduce the characteristics of each Yosakoi team, and many various dance processes have been generated at the festival. It is not easy to correct many samples for a partial process of each dance. According to the team’s situation of the Yosakoi festival, 679 data were prepared for the detection of the stops in the dance process.

To evaluate the stop detection accuracy, six methods (Comp. 1–Comp. 6) were used to compare the stop detection accuracy with that of the proposed model (PM). The outline of each method is shown in Table 4. Note that the batch size in the proposed method is set to 581 and the training rate is set to 0.001. The parameters of each comparative method were determined experimentally. Since long short-term memory (LSTM) [32] is analyzing series data in generally, the input data shapes in Comp. 2 and Comp. 3 are changed. Specifically, backwards and forwards are combined in the time direction for the velocity and acceleration features calculated from Equations (7)–(10), respectively. Moreover, the same preprocessing is performed on the time-series data. For this reason, the temporal window size of LSTM is 2, and the number of input nodes is half that of the NN. The effectiveness of feature data is examined by using time-series data. Specifically, the effectiveness of the NN at detecting stops is shown by using LSTM, which is said to be effective for time-series analysis. The effectiveness of the PM is shown below.

- The effectiveness of the feature data is confirmed by comparing the detection accuracy of the stops by the PM and Comp. 1.
- The effectiveness of stop detection by the NN is confirmed by comparing the detection accuracy of stops by the PM and Comp. 2.
- The effectiveness of combining feature data and NN in detecting stops is confirmed by comparing the accuracy of the PM and Comp. 3.
- We verify that the PM is more effective than traditional supervised learning-based methods by comparing the detection accuracy of stops by the PM and Comp. 4–Comp. 6.

Based on the previous section, 679 feature data are constructed from these dance motion data and used as training data for the PM, Comp. 2, and Comp. 4–Comp. 6. Moreover, in the velocity $v_{i}^{\mathrm{norm}}$ and acceleration $a_{i}^{\mathrm{fil}}$ in the previous section, let *t* be the stop possibility frame in Figure 4 and Figure 5. In this case, 679 time-series data consisting of $v_{i}^{\mathrm{norm}}$ and $a_{i}^{\mathrm{fil}}$ corresponding to the interval of t−25≤i≤t+25 are used as training data for Comp. 1 and Comp. 3. Moreover, the time-series data are divided into training data, verification data, and test data as shown in Table 5.

#### 4.1.2. Explanation of Evaluation Index

Next, the evaluation index is explained. In stop detection, it is important to detect a greater number of correct stops. For this reason, it is necessary to evaluate the recall for stop detection. On the other hand, it is also important to reduce false positives. Therefore, it is necessary to evaluate precision as well. For this reason, the F-measure, which is the harmonic mean of precision and recall, is used as the evaluation index for stop detection. The goal of the experiments is to detect stops, and it is impossible to classify short, normal, and long stops accurately, but this does not matter. Therefore, short, normal, and long stops are collectively referred to as stops. At this time, the F-measure is calculated by Equations (14)–(16).

(14) $$\begin{array}{ccc} \mathrm{Precision} & = & \frac{\mathrm{TP}}{\mathrm{TP}+\mathrm{FP}} \end{array}$$

(15) $$\begin{array}{ccc} \mathrm{Recall} & = & \frac{\mathrm{TP}}{\mathrm{TP}+\mathrm{FN}} \end{array}$$

(16) $$\begin{array}{ccc} \mathrm{F-measure} & = & \frac{2\times \mathrm{Precision}\times \mathrm{Recall}}{\mathrm{Precision}+\mathrm{Recall}} \end{array}$$

where, true positive (TP) is a set composed of the predictions (stops) and correct answers (stops); false positive (FP) is a set composed of the prediction (stops) and correct answer (no stop); and false negative (FN) is a set composed of the prediction (no stops) and correct answer (stops). The four stop classes are prepared in our detection method via NN architecture. According to our basic concept of detecting the stops in Yosakoi dance, the evaluation is based on two classes: no stop and stops.

#### 4.1.3. Results and Discussion

**Figure 8:**
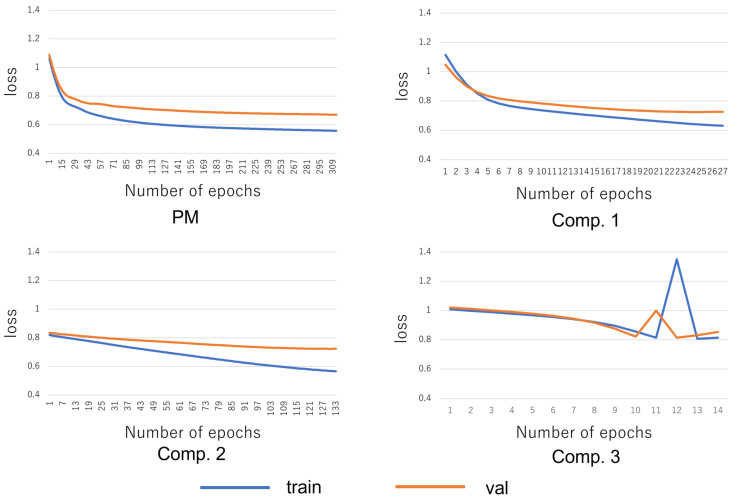
Transition of loss function.

The stop detection results of all methods are shown in Table 6. The accuracy is based on the value of the test data when the loss of the validation data stops decreasing. The transition of the loss function of the training data and the verification data in the NN-based methods is shown in Figure 8. Note that all data were recognized as no stop, so the precision and F-measure are not calculated in Comp. 3.

First, from Table 6, the F-measure of the PM stops is shown to be higher than those in Comp. 1–Comp. 6. From this, it can be said that the PM is effective in stop detection. Specifically, first, it can be seen that the PM has succeeded in improving the recall and F-measure when compared with Comp. 1. This shows the effectiveness of introducing feature data. Next, by comparing the PM with Comp. 2, it can be seen that the recall, precision, and F-measure have been successfully improved. This indicates the effectiveness of introducing the NN in stop detection. Next, by comparing the PM with Comp. 3, it can be seen that the recall, precision, and F-measure have been successfully improved. This indicates the effectiveness of combining feature data and the NN in stop detection. Moreover, by comparing the PM and Comp. 2 with Comp. 4–Comp. 6, it can be seen that the precision and F-measure have been successfully improved. This indicates the effectiveness of the NN-based method in stop detection. From the above, the effectiveness of the PM was shown. From Figure 8, it can be seen that the learning stopped before the tendency of the loss function of the verification data began to increase. From this, it can be said that overfitting was successfully suppressed. From Table 6, when feature data are used in the same machine learning method, the precision, recall, and F-measure tend to be higher than when time-series data are used. From this, it can be said that the feature data can express the difference between stops and no stops better than the time-series data. From the PM results, it can be concluded that the most accurate stop detection model has been constructed from among the tested methods.

### 4.2. Confirmation of Visualization Timing of Stops

In this section, the visualization timing of the detected stops is confirmed.

#### 4.2.1. Confirmation Method

The method by which we confirm the visualization accuracy of stops is explained. By applying the various methods used in the previous section to actual dance motion data, the visualization accuracy of the stops is evaluated. The dance motion data to be used in this experiment are 90 s of dance motion performed by another skilled dancer with 15 years of dance experience. This dance motion includes 32 stops (14 short, 15 normal, and 3 long stops). These dance motion data show that various models visualize only the stops performed by the right fist.

At this time, the number of times a correct stop is visualized is the number of detections, the number of times a no stop is visualized is the number of false positives, and the number of times a correct stop is not detected is the undetected number. The visualization accuracy of stops is quantitatively evaluated based on the number of detected stops, the number of false positives, and the number of undetected stops.

#### 4.2.2. Results and Discussion

**Figure 9:**
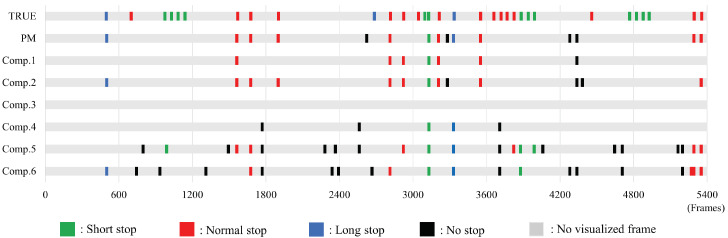
Comparative example of visualization timing of stops.

The visualization result of the stops is shown in Figure 9. However, the green part in the figure is a visualized short stop, the red part is a visualized normal stop, the blue part is a visualized long stop, the black part is a visualized no stop, and the gray part is a point in time where neither a stop nor a not stop was visualized. The numbers of stop detections, nondetections, and false positives are shown in Table 7.

First, Figure 9 and Table 7 show that the PM visualized more stops than Comp. 1–Comp. 4 and Comp. 6. Moreover, the number of false positives in the PM is less than that in Comp. 5. From this, we can judge that the PM is most effective in stop visualization. Then, the characteristics of the stops visualized in the PM are discussed. From Figure 9, a normal stop tends to be more easily visualized than a short or long stop. A normal stop has a longer stopping time than a short stop. These results suggest that the duration of a stop is a major factor in the criteria for judging a stop. In addition, the reason for the low visualization accuracy of a long stop is considered to be the insufficient number of data compared to the other labels.

Next, the false-positive behavior of the PM is discussed. When we checked the falsely detected motions, we found that folding motions such as waving tended to be falsely detected as stops. For example, consider a choreographed movement where the hands are waving. The apparent motion appears to stop only for a short time during the moment when the direction of the wave reverses. In particular, turning movements tended to be similar to those of short stops. From these points, it is considered that folding motions were mistakenly detected as short stops. However, considering the practical application of the stop detection model, the future challenge is how to increase recall while maintaining high precision. This problem is expected to be solved by increasing the number of high-quality data from people with dance experience.

Finally, we discuss aspects of different dance analysis research approaches. Table 8 shows the analysis and visualization functions in the related studies. As the table shows, all the methods perform movement analysis based on the characteristic elements of each dance. First, the proposed method and those in the literature [15,17,20] perform motion analysis based on features characteristic of a dance. In addition, the proposed method and those in the literature [13,14] provide motion analysis and training applications. From the above, it is important to link motion analysis and visualization technologies to develop from fundamental analysis to practical applications, such as dance practice and stage use.

## 5. Conclusions

In group dance, stops are important to improve the synchronization of the troupe’s movements. However, due to the effects of COVID-19, school, work, and so on, it is difficult to practice dancing in groups. This study proposes a dance practice support system based on detecting stops in a remote environment. The most accurate stop detection among machine learning methods was achieved by training the NN with velocity and acceleration features. Experiments showed the effectiveness of the proposed method. In addition, the detected stops were visualized using a humanoid 3D model. With these, remote control construction of a future dance practice environment was proposed.

In the experiment, the NN’s detected stops more accurately than comparative methods. On the other hand, the comparative methods also showed relatively high detection results. From this point of view, it can be said that it is possible to detect stops using various models, and it is expected that a system can be developed at low cost and have a direct effect on dance practice. In addition, because stops are an essential technique not only in Yosakoi Soran but also in a wide range of various genres of dance, the proposed system can be easily applied to other forms of dance by obtaining training data sets from other experienced dancers.

It is difficult to estimate 3D human pose with high accuracy, including depth, based on video images. Therefore, although MoCap was used in this study, the proposed method can be used if the coordinate information can be obtained in three dimensions, including depth. If video-based 3D human pose estimation technology including depth [36,37,38] can be put to practical use, a better training environment will be constructed. 

## Figures and Tables

**Table 1:** Stops characteristics.

Types of Stops	Details
Short stop	Operation stop time is about 25 frames.
Normal stop	Operation stop time is about 50 frames.
Long stop	Operation is stopped for a while.

**Table 2:** Details of each UI in visualization system.

UI	Details
Now Frame	Display the current frame.
Load (Student)	Read the dance movement data of the expert dancer.
Load (Teacher)	Read the performer’s dance movement data.
Start	Start playback of dance movement data.
Stop	Pause playback of dance movement data.
Finish	End playback of dance movement data.
0.5×	Play back dance movement data at 0.5× speed.
−0.5×	Rewind dance movement data at 0.5× speed.
1×	Play dance movement data at 1× speed
−1×	Rewind dance movement data at 1× speed
2×	Play back dance movement data at 2× speed.
−2×	Rewind dance movement data at 2× speed.

**Table 3:** Details of each dance movement.

Subject	Gender	Age	Height	Dance Experience	No. of Samples
A	Male	23 years old	164 cm	9 years	128
B1	Male	23 years old	168 cm	15 years	135
B2	Male	23 years old	168 cm	15 years	138
B3	Male	23 years old	168 cm	15 years	135
C	Female	36 years old	164 cm	19 years	143

**Table 4:** Overview of each method.

	Model	Data	Input Size	No. of Hidden Layers	No. of Hidden Nodes	Output Size
PM	NN	Feature data	4	1	16	4
Comp. 1	NN	Time-series data	102	1	128	4
Comp. 2	LSTM [32]	Feature data	2 × 2	1	16	4
Comp. 3	LSTM [32]	Time-series data	51 × 2	1	128	4
	**Model**	**Data**	**Input Size**	**Kernel**		**Output Size**
Comp. 4	Nonlinear Support Vector Machine [33]	Feature data	4	Radial basis function		4
	**Model**	**Data**	**Input Size**	**No. of Neighbors**		**Output Size**
Comp. 5	k-Nearest Neighbor [34]	Feature data	4	5		1
	**Model**	**Data**	**Input Size**	**No. of Trees in the Forest**		**Output Size**
Comp. 6	Random Forest [35]	Feature data	4	115		4

**Table 5:** Breakdown of the number of data sets for each training.

	Training	Verification	Testing
short stop	50	10	10
normal stop	51	12	12
long stop	9	3	3
no stop	363	78	78
Total	473	103	103

**Table 6:** Stop detection accuracy of each method.

	Precision	Recall	F-Measure
PM	0.938	**0.600**	**0.732**
Comp. 1	**1.000**	0.400	0.571
Comp. 2	0.813	0.520	0.634
Comp. 3	-	0	-
Comp. 4	0.698	0.539	0.546
Comp. 5	0.715	0.472	0.536
Comp. 6	0.641	0.575	0.558

**Table 7:** Quantitative evaluation of visualization accuracy.

	No. of Stop Detections	No. of Nondetections	No. of False Positives
PM	**11**	21	4
Comp. 1	6	26	1
Comp. 2	10	22	3
Comp. 3	0	32	0
Comp. 4	2	30	3
Comp. 5	**11**	21	12
Comp. 6	8	24	12

**Table 8:** List of analyses and visualization features in related studies.

Literature	No. of Subjects/Dance Genres	Analysis Examples	Application for Motion Visualization
PM	5 / Yosakoi (JPN)	NN-based stop detection	Highlighting a teacher and a student stop with VR
Chan et al. [13]	6 / Hip-hop and a-go-go (USA)	Motion matching from motion database	Highlighting incorrect movement joints with VR
Hachimura et al. [14]	5 / Street dance (USA)	-	Overlay of the computer graphics characteristics of a trainer with AR
Shiratori et al. [15]	2 / Aizu-bandaisan (JPN)	Segmentation of motion sequence based on the music rhythm	-
Yoshimura et al. [17]	5 / Fuji Musume (JPN)	Proposal of coordinate system considering local moving for motion tracking	-
Aristidou et al. [20]	3 / Bachatta dance (DMA)	Proposal of Laban Movement Analysis motion features for Laban	Only playback of tracked motion with VR

## Data Availability

Not applicable.

## Abbreviations

ICT	Information and Communication Technology
AI	Artificial Intelligence
MoCap	Motion capture
NN	Neural network
PN	Perception Neuron
ReLU	Rectified Linear Unit
VR	Virtual Reality
UI	User Interfaces
PM	Proposed method
LSTM	Long Short-Term Memory
TP	True Positive
FP	False Positive
FN	False Negative
